# Supplementary material for: The dynamic assembly of distinct RNA polymerase I complexes modulates rDNA transcription
Source: eLife. 2017 Mar 6;6:e20832. doi: 10.7554/eLife.20832 (PMC5362265; doi:10.7554/eLife.20832)
Supplement: Supplementary file 1. — DOI: http://dx.doi.org/10.7554/eLife.20832.027 [file elife-20832-supp1.docx]

**Supplementary file 1. Strains**

| **Construct** | **Strain** | **Ploidy** | **Genotype** | **Reference** |
| --- | --- | --- | --- | --- |
| wt | OGY0336 | n | *MATa, his3-200, leu2-3,112, ura3-52, lys2-801, tor1-1, fpr1::, TUB4-RFP-FKBP-natNT2,* | Laboratory collection |
| wt | OGY0350 | n | *MATa, his3-200, leu2-3,112, ura3-52, lys2-801, tor1-1, fpr1::, TUB4-RFP-FKBP-natNT2, RPA190-FRB-hphNT1,* | Laboratory collection |
| wt | OGY0354 | n | *MATa, his3-200, leu2-3,112, ura3-52, lys2-801, tor1-1, fpr1::, TUB4-RFP-FKBP-natNT2, RPA190-3xmyeGFP-kanMX4* | Laboratory collection |
| wt/wt | OGY0362 | 2n | *MATa/alpha, his3-200/ his3-200, leu2-3,112/ leu2-3,112, ura3-52/ ura3-52, lys2-801/ lys2-801, tor1-1/ tor1-1, fpr1::/ fpr1::, TUB4-RFP-FKBP-natNT2/ TUB4-RFP-FKBP-natNT2, RPA190-FRB-hphNT1, RPA190-3xmyeGFP-kanMX4, RPA43 / RPA43* | Laboratory collection |
| NIC96-RFP | OGY0346 | 2n | *MATa/alpha, his3-200/ his3-200, leu2-3,112/ leu2-3,112, ura3-52/ ura3-52, lys2-801/ lys2-801, tor1-1/ tor1-1, fpr1::/ fpr1::, NIC96-RFP-natNT2/ NIC96, RPA190-FRB-myeGFP- kanMX4/RPA190* | Laboratory collection |
| wt | OCG0150 | n | *MATa, his3-200, leu2-3,112, ura3-52, lys2-801, tor1-1, fpr1::, TUB4-RFP-FKBP-natNT2, RPA190-FRB-hphNT1, RRN3-3xmyeGFP-kanMX4, RPA43* | Laboratory collection |
| ΔA14 | OCG0176 | n | *MATa, his3-200, leu2-3,112, ura3-52, lys2-801, tor1-1, fpr1::, TUB4-RFP-FKBP-natNT2, RPA190-FRB-hphNT1, RRN3-3xmyeGFP-kanMX4, rpa14::HIS3* | Laboratory collection |
| A14ΔCt | OCG0177 | n | *MATa, his3-200, leu2-3,112, ura3-52, lys2-801, tor1-1, fpr1::, TUB4-RFP-FKBP-natNT2, RPA190-FRB-hphNT1, RRN3-3xmyeGFP-kanMX4, rpa14ΔP101-stop::HIS3* | Laboratory collection |
| ΔA14+A14 | OCG0215 | n | *MATa, his3-200, leu2-3,112, ura3-52, lys2-801, tor1-1, fpr1::, TUB4-RFP-FKBP-natNT2, RPA190-FRB-hphNT1, RRN3-3xmyeGFP-kanMX4, rpa14::HIS3 [pRS315-RPA14]* | Laboratory collection |
| ΔA14+A14ΔTAloop | OCG0207 | n | *MATa, his3-200, leu2-3,112, ura3-52, lys2-801, tor1-1, fpr1::, TUB4-RFP-FKBP-natNT2, RPA190-FRB-hphNT1, RRN3-3xmyeGFP-kanMX4, rpa14::HIS3 [pRS315-rpa14ΔS53-N77]* | Laboratory collection |
| ΔA14+empty vector | OCG0206 | n | *MATa, his3-200, leu2-3,112, ura3-52, lys2-801, tor1-1, fpr1::, TUB4-RFP-FKBP-natNT2, RPA190-FRB-hphNT1, RRN3-3xmyeGFP-kanMX4, rpa14::HIS3 [pRS315]* | Laboratory collection |
| ΔA14+A14/ ΔA14+A14 | OCG0278 | 2n | *MATa/alpha, his3-200/ his3-200, leu2-3,112/ leu2-3,112, ura3-52/ ura3-52, lys2-801/ lys2-801, tor1-1/ tor1-1, fpr1::/ fpr1::, TUB4-RFP-FKBP-natNT2/ TUB4-RFP-FKBP-natNT2, RPA190-FRB-hphNT1, RPA190-3xmyeGFP-kanMX4, rpa14::HIS3/ rpa14::HIS3 [pRS315-RPA14]* | Laboratory collection |
| ΔA14+A14ΔTAloop | OCG0279 | 2n | *MATa/alpha, his3-200/ his3-200, leu2-3,112/ leu2-3,112, ura3-52/ ura3-52, lys2-801/ lys2-801, tor1-1/ tor1-1, fpr1::/ fpr1::, TUB4-RFP-FKBP-natNT2/ TUB4-RFP-FKBP-natNT2, RPA190-FRB-hphNT1, RPA190-3xmyeGFP-kanMX4, rpa14::HIS3/ rpa14::HIS3 [pRS315-rpa14ΔS53-N77]* | Laboratory collection |
| Pil-RFP | OGY0297 | n | *MATα, his3Δ1 leu2Δ0 ura3Δ0 LYS+, can1::STE2pr-LEU2, lyp1::, tor1-1, fpr1::klURA, STE5-3xmyeGFP-kanMX4, STE11-FRB-hphNT1, Pil1-RFP-FKBP-natNT2* | Laboratory collection |
| Tub4-RFP | OGY0300 | n | *MATα, his3Δ1 leu2Δ0 ura3Δ0 LYS+, can1::STE2pr-LEU2, lyp1::, tor1-1, fpr1::klURA, STE5-3xmyeGFP-kanMX4, STE11-FRB-hphNT1, TUB4-RFP-FKBP-natNT2* | Laboratory collection |
| C160F/C128G | OGY0615 | 2n | *MATa/alpha, his3-200/ his3-200, leu2-3,112/ leu2-3,112, ura3-52/ ura3-52, lys2-801/ lys2-801, tor1-1/ tor1-1, fpr1::/ fpr1::, TUB4-RFP-FKBP-natNT2/ TUB4-RFP-FKBP-natNT2, RPC1/RPC1-FRB-hphNT1, RPC2/RPC2-3xmyeGFP-kanMX4,* | Laboratory collection |
| C160F/C160G | OGY0614 | 2n | *MATa/alpha, his3-200/ his3-200, leu2-3,112/ leu2-3,112, ura3-52/ ura3-52, lys2-801/ lys2-801, tor1-1/ tor1-1, fpr1::/ fpr1::, TUB4-RFP-FKBP-natNT2/ TUB4-RFP-FKBP-natNT2, RPC1-FRB-hphNT1/ RPC1-3xmyeGFP-kanMX4,* | Laboratory collection |
| Rpb3F/Rpb1G | OGY0618 | 2n | *MATa/alpha, his3-200/ his3-200, leu2-3,112/ leu2-3,112, ura3-52/ ura3-52, lys2-801/ lys2-801, tor1-1/ tor1-1, fpr1::/ fpr1::, TUB4-RFP-FKBP-natNT2/ TUB4-RFP-FKBP-natNT2, RPB3/RPB3-FRB-hphNT1, RPB1/RPB1-3xmyeGFP-kanMX4,* | Laboratory collection |
| Rpb3R/Rpb3G | OGY0619 | 2n | *MATa/alpha, his3-200/ his3-200, leu2-3,112/ leu2-3,112, ura3-52/ ura3-52, lys2-801/ lys2-801, tor1-1/ tor1-1, fpr1::/ fpr1::, TUB4-RFP-FKBP-natNT2/ TUB4-RFP-FKBP-natNT2, RPB3-FRB-hphNT1/ RPB3-3xmyeGFP-kanMX4,* | Laboratory collection |
| A43_307Stop | OGY0693 | n | *MATa, his3-200, leu2-3,112, ura3-52, lys2-801, tor1-1, fpr1, TUB4-RFP-FKBP-natNT2, RPA190-FRB-hphNT1, RRN3-3xmyeGFP-kanMX4, RPA43_307Stop* | Laboratory collection |
| A43_307Stop/ A43_307Stop | OGY0698 | 2n | *MATa/alpha, his3-200/ his3-200, leu2-3,112/ leu2-3,112, ura3-52/ ura3-52, lys2-801/ lys2-801, tor1-1/ tor1-1, fpr1::/ fpr1::, TUB4-RFP-FKBP-natNT2/ TUB4-RFP-FKBP-natNT2, RPA190-FRB-hphNT1, RPA190-3xmyeGFP-kanMX4, RPA43_307Stop / RPA43_307Stop* | Laboratory collection |
| ΔA14+A14Δα2-Ct | OCG0231 | n | *MATa, his3-200, leu2-3,112, ura3-52, lys2-801, tor1-1, fpr1, TUB4-RFP-FKBP-natNT2, RPA190-FRB-hphNT1, RRN3-3xmyeGFP-kanMX4,* | Laboratory collection |
| ΔA14+A14-R91E | OCG0232 | n | *MATa, his3-200, leu2-3,112, ura3-52, lys2-801, tor1-1, fpr1, TUB4-RFP-FKBP-natNT2, RPA190-FRB-hphNT1, RRN3-3xmyeGFP-kanMX4,* *rpa14::HIS3 [pRS315-RPA14-R91E]* | Laboratory collection |
| \| YPH500 \| \| --- \| \|  \| |  | n | *MATα, ura3-52, lys2-801_amber , ade2-101_ochre, trp1-Δ63, his1-Δ200, leu2-Δ1, rpa43::kan^r^, rrn3::his5+, [pGEN]* | Laferté, G&D 2006 |
| CARA |  | n | *MATα, ura3-52, lys2-801_amber , ade2-101_ochre, trp1-Δ63, his1-Δ200, leu2-Δ1, rpa43::kan^r^, rrn3::his5+, [pGEN-RRN3-RPA43]* | Laferté, G&D 2006 |
| A190-TAP/A190MYC | OCG0235 | 2n | *Mata/α ADE2/ade2-1, his3-∆1/his3-11, leu2-∆0/leu2-,112, TRP1/trp1-1, met15-∆0 ura3-∆0/ ura3-1, RPA190-TAP::HIS3/RPA190-MYC kanMX6* | Laboratory collection |
