# Supplementary material for: The dynamic assembly of distinct RNA polymerase I complexes modulates rDNA transcription
Source: eLife. 2017 Mar 6;6:e20832. doi: 10.7554/eLife.20832 (PMC5362265; doi:10.7554/eLife.20832)
Supplement: Supplementary file 2. — DOI: http://dx.doi.org/10.7554/eLife.20832.028 [file elife-20832-supp2.docx]

**Supplementary file 2. Cryo-EM structure statistics**

| Pixel size (Å/pixel) | 1.77 | | |
| --- | --- | --- | --- |
| Number of grids | 1 | | |
| Days of data collection | 1 | | |
| All micrographs | 1,288 | | |
| Selected micrographs | 1,171 | | |
| Particles after 2D-class | 190,722 | | |
| Particle subsets | **Pol I–Rrn3** | **Apo-Pol I (high)^1^** | **Apo-Pol I** |
| Final number of particles | 32,175 | 122,348 | 90,173 |
| Resolution (Å) | 7.7 | 4.9 | 5.6 |
| AccuracyRotations (º) | 3.87 | 3.01 | 3.18 |
| AccuracyTranslations (pixel) | 1.72 | 1.21 | 1.28 |
| Estimated B-factor (Å^2^) | -537 | -111 | -160 |

^1^Apo-Pol I (high) includes the sum of particles from the other two groups
